# Comparative Analysis of Ketorolac and Parecoxib for Postoperative Pain Management in Uvulopalatopharyngoplasty

**DOI:** 10.3390/jcm13154422

**Published:** 2024-07-28

**Authors:** Cheng-Yu Hsieh, Chuan-Hung Sun, Chung-Ching Lin, Yi-Fan Chou

**Affiliations:** 1 Department of Otolaryngology, Head and Neck Surgery, Taichung Tzu Chi Hospital, Buddhist Tzu Chi Medical Foundation, Taichung 427213, Taiwan; chengyu384650@gmail.com (C.-Y.H.); sunch7297@gmail.com (C.-H.S.); a0931301587@gmail.com (C.-C.L.); 2 School of Medicine, Tzu Chi University, Hualien 970374, Taiwan

**Keywords:** parecoxib, ketorolac, uvulopalatopharyngoplasty, pain control

## Abstract

**Background/Objectives:** Uvulopalatopharyngoplasty (UPPP) is a prevalent surgical procedure for treating obstructive sleep apnea. Effective postoperative pain management is crucial for patient comfort and recovery. This study aimed to compare the analgesic efficacies of parecoxib and ketorolac in patients undergoing UPPP. **Methods:** A prospective, randomized, double-blind study was conducted on 83 patients who received either parecoxib (40 mg intravenously every 12 h) or ketorolac (30 mg intravenously every 8 h) for 2 days following UPPP. Postoperative pain and swallowing discomfort were assessed using visual analog scales (VASs) at 4, 24, 48, and 72 h. The time to resume eating and adverse reactions were also recorded. **Results:** At 24 and 48 h postoperatively, the mean VAS score was significantly higher in the ketorolac group compared to the parecoxib group (5.0 ± 2.3 vs. 3.6 ± 2.2, *p* = 0.005 and 3.9 ± 2.2 vs. 2.5 ± 1.7, *p* < 0.001, respectively). However, no significant difference in the mean VAS scores was observed between the two groups at 72 h postoperatively. With regards to postoperative swallowing pain, the ketorolac group exhibited significantly higher mean VAS scores than the parecoxib group at 4, 24, 48, and 72 h postoperatively. **Conclusions:** Intravenous parecoxib may offer superior analgesic benefits in the early postoperative period, particularly in alleviating swallowing pain, compared to ketorolac in UPPP procedures.

## 1. Introduction

Uvulopalatopharyngoplasty (UPPP) is a commonly performed surgical procedure for treating mild or moderate obstructive sleep apnea. The surgery involves shortening the uvula and soft palate, as well as performing a tonsillectomy. Despite advancements in anesthesia and surgical techniques, postoperative pharyngeal pain continues to affect patient comfort and recovery. Swallowing pain post-surgery is a significant concern because it can result in reduced oral intake, dehydration, sleep disturbances, and prolonged hospital stays [1,2]. Therefore, effective pain management is essential to optimize postoperative outcomes and ensure patient satisfaction.

Ketorolac, a nonsteroidal anti-inflammatory drug (NSAID), is widely used in clinical practice to manage pain and inflammation [3]. Ketorolac is frequently used in perioperative, acute, and chronic pain management to provide effective analgesia and minimize reliance on opioids [3]. Opioids may lead to respiratory depression, which can be life-threatening in patients with obstructive sleep apnea syndrome [4]. Due to its non-opioid nature, ketorolac offers an alternative option for pain relief, reducing reliance on opioids and potentially minimizing opioid-related adverse effects. Ketorolac can be administered intravenously or intramuscularly to inhibit the production of prostaglandins [3], thereby reducing pain and inflammation post-surgery. The drug is commonly used in orthopedic, dental, and gynecological surgeries. However, the adverse effects of NSAIDs, such as gastrointestinal bleeding, ulceration, edema, hypertension, and acute renal failure, are associated with the inhibition of cyclooxygenase-1 (COX-1) in the gastric mucosa and kidneys [5]. To mitigate these risks, cyclooxygenase-2 (COX-2) specific inhibitors have been developed to relieve pain and inflammation through selective COX-2 inhibition [6].

Parecoxib, a selective COX-2 inhibitor, offers effective postoperative analgesia without inducing respiratory depression [7]. Its mechanism of action involves inhibiting COX-2-mediated prostaglandin synthesis, which reduces the production of pain and inflammatory mediators [8]. By selectively inhibiting both peripheral and central COX-2, parecoxib demonstrates a rapid onset and prolonged duration of action [9]. Parecoxib has become integral to multimodal analgesia protocols in various surgical specialties such as abdominal, orthopedic, thoracic, and spinal surgeries [10,11].

Both parecoxib and ketorolac have advantages, such as rapid onset, suitable duration of action, and easy accessibility. However, to date, a paucity of direct, comparative, and randomized controlled trials to establish a consensus on the optimal choice in UPPP exists. Therefore, this study aimed to compare the clinical effects of parecoxib and ketorolac in patients undergoing UPPP. The primary outcome measure in this study was the postoperative pain score, which was assessed using the visual analog scale (VAS) at 4, 24, 48, and 72 h post-surgery. The secondary outcome measures included the incidence of postoperative complications. This included the occurrence of nausea, vomiting, abdominal discomfort, pruritus, and secondary bleedings within 3 weeks post-surgery. By elucidating the comparative effectiveness of parecoxib and ketorolac in UPPP, our findings aim to provide valuable insights for clinicians when selecting the most appropriate analgesic regimen.

## 2. Materials and Methods

### 2.1. Ethical Consideration

This prospective randomized controlled study was approved by the appropriate research ethics committee (REC-11041) and adhered to the Declaration of Helsinki guidelines. Written informed consent was obtained from all enrolled patients, following a protocol approved by the relevant research ethics committee.

### 2.2. Experimental Design

Eighty-three patients aged 20–70 years were prospectively enrolled for UPPP in tertiary referral centers between August 2021 and August 2023. All participants fulfilled the American Academy of Otolaryngology Head and Neck Surgery criteria for chronic or recurrent tonsillitis, or tonsillar hypertrophy with obstructive symptoms. Patients were excluded if they were pregnant, had severe arrhythmias or tonsillar cancer, underwent combination surgeries, were allergic to NSAIDs, had severe underlying diseases such as cardiovascular disease, or had a bleeding tendency.

Patients were randomly assigned to either the ketorolac or parecoxib group. The parecoxib group (*n* = 42) received a 40 mg intravenous injection of parecoxib 30 min before completing UPPP, followed by subsequent 40 mg intravenous doses of parecoxib every 12 h for 2 days, resulting in a cumulative dose of 160 mg. The ketorolac group (*n* = 41) received a 30 mg intravenous injection of ketorolac 30 min before completing UPPP, followed by subsequent 30 mg intravenous doses of ketorolac every 8 h for 2 days, amounting to a cumulative dose of 180 mg.

All surgical procedures were performed under general anesthesia by the same surgeon using electrocautery dissection. A local anesthetic injection of 2 cc lidocaine over the peritonsillar area was administered preoperatively to reduce pain by blocking peripheral nociceptive excitation [12]. During tonsillectomy, cotton balls soaked with 1% bosmin were tightly packed into the tonsillar fossa for hemostasis of mucosal bleeding [13]. Bipolar electrocauterization was used for hemostasis if persistent active bleeding was uncontrolled. Opioids were avoided due to their potential for nausea and respiratory inhibition [14]. Postoperative resting pharyngeal and swallowing pain were assessed using the visual analog scale (VAS), with a score of 0 indicating no pain and 10 indicating maximum pain. The VAS assessments were conducted at fixed intervals postoperatively: 4, 24, 48, and 72 h post-UPPP by a physician who was blinded to the group allocation. These assessments were scheduled immediately before the subsequent dose of either medication to ensure that the measurements reflect the trough levels of drug efficacy. All patients were also blinded to their treatment allocation. Postoperative data regarding nausea, vomiting, fever, time to oral intake, and bleeding events were collected. All patients received a standardized dose of acetaminophen, 500 mg, three times daily for the first 72 h postoperatively. Generally, patients were discharged 3 days postoperatively after an examination that confirmed the surgical wound was uneventful and was not oozing.

### 2.3. Statistical Analysis

Data on postoperative resting pharyngeal pain, swallowing pain, and adverse events were collected and analyzed. Descriptive statistics are presented as the means and standard deviations for continuous variables, while categorical variables are expressed as counts and percentages. The study had a statistical effect size of 70% and a power of 80%. The Mann–Whitney U Test was used to evaluate the postoperative resting pharyngeal pain scores, and postoperative swallowing pain scores. All statistical analyses were conducted using SPSS 20.0 statistical software, with statistical significance set at *p* < 0.05.

## 3. Result

### 3.1. Patient Characteristics

A total of 83 patients were included in this study, and no instances of postoperative secondary bleeding occurred within three weeks post-surgery. Table 1 displays the demographic information of the patients; postoperative pain scores at 4, 24, 48, and 72 h after UPPP; and the adverse effects observed in each group.

### 3.2. Primary Outcomes

#### 3.2.1. Postoperative Resting Pharyngeal Pain

The mean VAS score for postoperative resting pharyngeal pain at 4 h postoperatively was 6.4 ± 2.3 in the ketorolac group and 5.4 ± 2.5 in the parecoxib group (Table 2). At 24 h postoperatively, the ketorolac group had a significantly higher mean VAS score compared to the parecoxib group (Table 2). Similarly, at 48 h postoperatively, the ketorolac group also showed a significantly higher mean VAS score compared to the parecoxib group (Table 2). However, no significant difference was observed in the mean VAS score at 72 h postoperatively between the two groups (Table 2).

**Figure 1:**
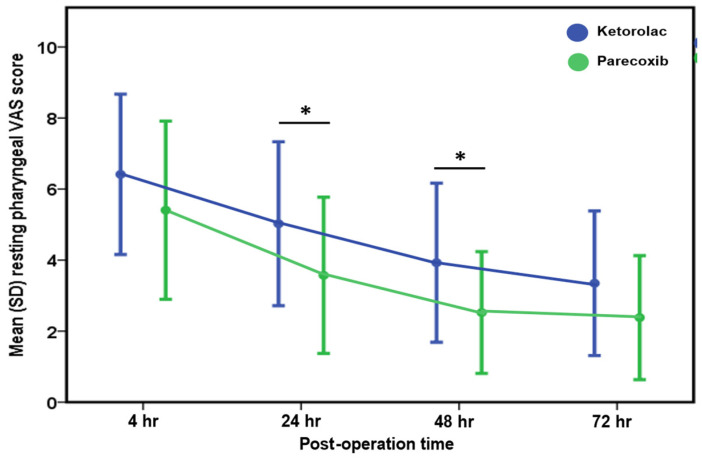
Comparison of resting pharyngeal VAS score postoperatively between the ketorolac and parecoxib groups. * indicates a statistically significant difference between two groups. * *p* < 0.05.

As shown in Figure 1, the mean VAS scores for resting pharyngeal pain were consistently higher in the ketorolac group compared to the parecoxib group at 24 and 48 h postoperatively, indicating that parecoxib provided better pain relief during the early postoperative period.

#### 3.2.2. Postoperative Swallowing Pain

**Figure 2:**
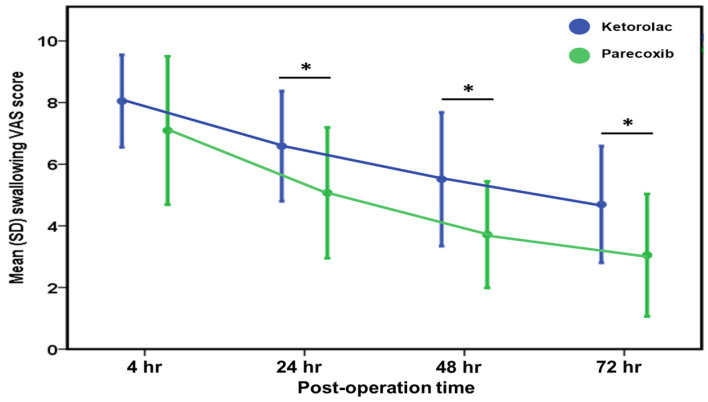
Comparison of swallowing VAS score postoperatively between the ketorolac and parecoxib groups. * indicates a statistically significant difference between two groups. * *p* < 0.05.

The mean VAS score for postoperative swallowing pain at 4 h postoperatively was 8.0 ± 1.5 in the ketorolac group and 7.1 ± 2.4 in the parecoxib group (*p* = 0.113; Table 2). At 24 h postoperatively, the ketorolac group exhibited a significantly higher mean VAS score compared to the parecoxib group (6.6 ± 1.8 vs. 5.1 ± 2.1, *p* = 0.001; Table 2). Similarly, at the 48 h mark post-surgery, the ketorolac group continued to display a significantly higher mean VAS score than the parecoxib group (5.5 ± 2.2 vs. 3.7 ± 1.7, *p* < 0.001; Table 2). Furthermore, at 72 h postoperatively, the mean VAS score remained notably higher in the ketorolac group compared to the parecoxib group (4.7 ± 1.9 vs. 3.1 ± 2.0, *p* = 0.013; Table 2). As illustrated in Figure 2, the parecoxib group consistently reported lower mean VAS scores for swallowing pain at all postoperative time points compared to the ketorolac group, highlighting the superior efficacy of parecoxib in managing postoperative swallowing pain.

### 3.3. Secondary Outcomes

#### 3.3.1. Incidence of Side Effects

Table 3 presents the incidence of postoperative complications in the two groups. No statistically significant differences in the incidences of nausea, vomiting, abdominal pain, or pruritus between the two groups were observed.

#### 3.3.2. Time to Resume Diet

The time to resume diet, measured as the number of hours from surgery completion to the oral intake of regular food, did not differ significantly between the two groups (*p* = 0.314; Table 3).

## 4. Discussion

### 4.1. Superiority of Parecoxib in Swallowing Pain Relief

To the best of our knowledge, this is the first study to compare the efficacy of two NSAIDs, parecoxib and ketorolac, in managing postoperative pain in patients undergoing UPPP. Our findings strongly suggest that parecoxib demonstrates superior efficacy over ketorolac in postoperative pain management, particularly in alleviating swallowing discomfort during the initial 3 days following UPPP (Figure 1).

Firstly, our results indicate that at 24 h post-surgery, patients who received parecoxib reported significantly lower mean VAS scores for swallowing pain compared to those who received ketorolac. This early advantage in pain relief with parecoxib is clinically significant, as it corresponds to the immediate postoperative recovery period when patients commonly experience discomfort while swallowing due to surgical trauma and tissue inflammation. Secondly, the superiority of parecoxib over ketorolac persisted at 48 h postoperatively. Patients in the ketorolac group continued to exhibit significantly higher mean VAS scores for swallowing pain than those in the parecoxib group. This sustained benefit of parecoxib reinforces its effectiveness in providing prolonged relief from distressing swallowing pain, which can significantly affect the patient’s overall postoperative experience and recovery [15]. Finally, while no significant difference in the mean VAS scores was observed between the two groups at 72 h postoperatively, it is noteworthy that parecoxib consistently maintained a lower mean VAS score, albeit without statistical significance. This trend suggests that the advantages of parecoxib in terms of swallowing pain relief may extend beyond the initial 48 h period.

### 4.2. Parecoxib: Optimal Pain Relief, Gastrointestinal Safety, and Drug Compatibility

NSAIDs, particularly nonselective ones that inhibit both COX-1 and COX-2, play a crucial role in managing perioperative pain. However, concerns about increased bleeding, especially in the gastrointestinal tract and surgical sites, have limited their widespread use among clinicians. This hesitancy stems from the dual inhibition of COX-1 [16], which affects platelet function and potentially leads to increased perioperative bleeding [5,16]. Parecoxib, a highly selective COX-2 inhibitor, has shown promising analgesic effects by reducing morphine requirements after surgeries like tonsillectomy [9]. COX-2 inhibitors selectively inhibit the COX-2 pathway, thereby reducing the degree of pain and inflammation [6,9]. However, patients undergoing UPPP often experience severe swallowing pain, which may be related to local inflammation of the wound and pharyngeal muscle tension caused by swallowing.

Parecoxib does not interfere with platelet aggregation, making it a suitable option for patients with coagulation-related concerns [7,17]. Unlike ketorolac, parecoxib reduces the risk of abdominal discomfort, ulcers, and bleeding [18]. This distinct advantage provides a safer and more tolerable postoperative experience for patients, particularly those who are prone to gastric complications. Parecoxib is contraindicated in patients allergic to parecoxib or other NSAIDs; those with a history of asthma, urticaria, or allergic reactions after taking aspirin; and in patients with active peptic ulcer or gastrointestinal bleeding. Additionally, parecoxib should be used with caution in patients with severe renal impairment, cardiovascular disease, or those who are pregnant or breastfeeding. In our study, the lack of significant differences observed between the groups can be attributed to the small sample size and short duration of usage. Although parecoxib has demonstrated superior analgesic efficacy, acknowledging the possibility of vomiting associated with its use is crucial. This aspect warrants further investigation and careful consideration in clinical decision-making.

### 4.3. Implications for Clinical Practice

In patients with obstructive sleep apnea syndrome, the choice of postoperative analgesics is critical to avoid excessive sedation and respiratory depression, which can exacerbate respiratory tract obstruction and pose life-threatening risks. The American Society of Anesthesiologists recommends local anesthesia for postoperative pain management in UPPP procedures to mitigate the adverse effects associated with systemic opioid use [14]. Given the inherent risks associated with opioids in these patients, the use of effective NSAIDs has been emphasized in clinical practice. This study demonstrated that parecoxib, administered twice daily, exhibited superior efficacy compared to ketorolac, administered thrice daily. However, the cost difference between parecoxib and ketorolac is significant. A 160 mg dose of parecoxib costs between $100 and $140, compared to $12 to $18 for a 180 mg dose of ketorolac. Although parecoxib offers certain clinical benefits, its higher price must be carefully considered.

The rapid onset and extended duration of action of parecoxib, alongside its compatibility with other commonly used anesthetics such as midazolam, propofol, and fentanyl, minimize potential drug interactions that might complicate postoperative pain management [19,20]. Despite the insights gained from this study, certain limitations warrant consideration. First, the relatively small sample size employed in our investigation may limit the generalizability of our findings. Second, the 72 h postoperative follow-up period may not adequately capture the long-term effects of parecoxib and ketorolac on pain relief and recovery.

## 5. Conclusions

Our study highlights the notable superiority of parecoxib over ketorolac in providing effective pain relief, particularly in the context of managing postoperative swallowing pain during the first 3 days following UPPP. The sustained effectiveness of parecoxib, coupled with its favorable gastrointestinal safety profile and drug compatibility, makes it a valuable choice for clinicians in optimizing pain management strategies for patients undergoing UPPP. These findings have important implications for improving the overall patient experience, emphasizing the importance of selecting appropriate analgesic regimens to enhance patient satisfaction and improve clinical outcomes in UPPP procedures.

## Figures and Tables

**Table 1:** Demographic and baseline characteristics of patients.

	Ketorolac (N = 41)	Parecoxib (N = 42)	*p*-Value
Sex			0.716
Female (n:%)	15 (36.6%)	17 (40.5%)	
Male (n:%)	26 (63.4%)	25 (59.5%)	
Age (yr.)	39.6 ± 13.4	41.7 ± 14.3	0.591
Height (cm)	121.7 ± 74.1	104.1 ± 81.6	0.431
Body weight (kg)	76.7 ± 16.3	75.4 ± 15.0	0.785
Body mass index	27.8 ± 5.1	27.1 ± 4.2	0.771

Data are presented as the median values and mean and standard deviation (SD). The Mann–Whitney U Test was used for continuous variables. The Chi-square test was used for categorical variables. *p* < 0.05.

**Table 2:** Between-group comparison of postoperative pain score.

	Ketorolac (N = 41)	Parecoxib (N = 42)	*p*-Value
Postoperative resting pharyngeal pain			
VAS, 4 h	6.4 ± 2.3	5.4 ± 2.5	0.090
VAS, 24 h	5.0 ± 2.3	3.6 ± 2.2	0.005 *
VAS, 48 h	3.9 ± 2.2	2.5 ± 1.7	<0.001 *
VAS, 72 h	3.3 ± 2.0	2.4 ± 1.7	0.107
Postoperative swallowing pain			
VAS, 4 h	8.0 ± 1.5	7.1 ± 2.4	0.108
VAS, 24 h	6.6 ± 1.8	5.1 ± 2.1	0.001 *
VAS, 48 h	5.5 ± 2.2	3.7 ± 1.7	<0.001 *
VAS, 72 h	4.7 ± 1.9	3.1 ± 2.0	0.010 *

Data are presented as the median values and mean and standard deviation (SD). The Mann–Whitney U Test was used for continuous variables. * *p* < 0.05

**Table 3:** Incidence of side effects in parecoxib and ketorolac groups.

	Ketorolac (N = 41)	Parecoxib (N = 42)	*p*-Value
Nausea	9 (22%)	8 (19%)	0.743
Vomiting	5 (12%)	10 (24%)	0.169
Abdominal discomfort	5 (12%)	2 (4.8%)	0.223
Pruritus	2 (4.9%)	3 (7.1%)	1.000
Time to resume soft diet			0.314
Operative day (n:%)	5 (12.2%)	5 (11.9%)	
Postoperative 24 h	18 (43.9%)	26 (61.9%)	
Postoperative 48 h	14 (34.1%)	8 (19.0%)	
Postoperative 72 h	4 (9.8%)	2 (4.8%)	
Postoperative 96 h	0 (0.0%)	1 (2.4%)	

Data are presented as number of patients (N) and percentages (%). The Chi-square test was used for categorical variables. The *p*-value represents the overall comparison between the two groups.

## Data Availability

The data presented in this study are available on request from the corresponding author. The data are not publicly available due to privacy or research ethics.
